# Health risk assessment and source apportionment of PM_2.5_-bound toxic elements in the industrial city of Siheung, Korea

**DOI:** 10.1007/s11356-022-20462-0

**Published:** 2022-05-04

**Authors:** Young Su Lee, Young Kwon Kim, Eunhwa Choi, Hyeri Jo, Hyeseung Hyun, Seung-Muk Yi, Jae Young Kim

**Affiliations:** 1 Department of Civil and Environmental Engineering, Seoul National University, 1 Gwanak-ro, Gwanak-gu, Seoul, Republic of Korea; 2 Department of Environmental Health Sciences, Graduate School of Public Health, Seoul National University, 1 Gwanak-ro, Gwanak-gu, Seoul, Republic of Korea; 3 Division of Policy Research, Green Technology Center, Seoul, 04554 Republic of Korea; 4 Institute of Construction and Environmental Engineering, Seoul National University, Gwanak-ro, Gwanak-gu, Seoul, Republic of Korea; 5 College of Environmental Design, University of California, Berkeley, Berkeley, CA USA

**Keywords:** Fine particulate matter, Source apportionment, Positive matrix factorization, Health risk assessment, Carcinogenic risk

## Abstract

The emission sources and their health risks of fine particulate matter (PM_2.5_) in Siheung, Republic of Korea, were investigated as a middle-sized industrial city. To identify the PM_2.5_ sources with error estimation, a positive matrix factorization model was conducted using daily mean speciated data from November 16, 2019, to October 2, 2020 (95 samples, 22 chemical species). As a result, 10 sources were identified: secondary nitrate (24.3%), secondary sulfate (18.8%), traffic (18.8%), combustion for heating (12.6%), biomass burning (11.8%), coal combustion (3.6%), heavy oil industry (1.8%), smelting industry (4.0%), sea salts (2.7%), and soil (1.7%). Based on the source apportionment results, health risks by inhalation of PM_2.5_ were assessed for each source using the concentration of toxic elements portioned. The estimated cumulative carcinogenic health risks from the coal combustion, heavy oil industry, and traffic sources exceeded the benchmark, 1E-06. Similarly, carcinogenic health risks from exposure to As and Cr exceeded 1E-05 and 1E-06, respectively, needing a risk reduction plan. The non-carcinogenic risk was smaller than the hazard index of one, implying low potential for adverse health effects. The probable locations of sources with relatively higher carcinogenic risks were tracked. In this study, health risk assessment was performed on the elements for which mass concentration and toxicity information were available; however, future research needs to reflect the toxicity of organic compounds, elemental carbon, and PM_2.5_ itself.

**Supplementary Information:**

The online version contains supplementary material available at 10.1007/s11356-022-20462-0.

## Introduction

Fine particulate matter (PM_2.5_) in the atmosphere is classified as a group 1 carcinogen by the World Health Organization (WHO) owing to its carcinogenicity to humans (WHO 2005; Anderson 2009). In many countries, PM_2.5_ concentration is used as a major indicator of air quality, and significant efforts have been made to reduce PM_2.5_ pollution (Riojas-Rodríguez et al. 2016; Nazarenko et al. 2021). For a proper PM_2.5_ management, pollution sources should be accurately managed by determining the relationship between the source characteristics and atmospheric concentrations (Kim et al. 2019; Fang et al. 2020; Long et al. 2021). However, when PM_2.5_ is released into the atmosphere, it immediately goes through complex mechanisms such as advection, diffusion, reaction, and deposition; therefore, it is difficult to identify its source (Anderson 2009; Riojas-Rodríguez et al. 2016). Thus, to effectively clarify the mechanisms and characteristics of PM_2.5_ pollution and improve air quality, scientific methods should be applied to identify and quantify PM_2.5_ sources (Wang et al. 2012; Belis et al. 2013; Hopke 2016). In addition, as the impacts on human health vary according to PM_2.5_ source, management priorities should be defined based on the evaluation of health impacts and source apportionment (Yang et al. 2013; Kim et al. 2015).

Receptor models based on chemical mass balance and principal component analysis as a statistical method have been widely used to identify PM_2.5_ sources (Samara et al. 2003; Choi et al. 2013; Yang et al. 2013). Karagulian et al. (2015) have reported a total of 419 source apportionment studies conducted in 51 countries around the world. Among the principal component analysis methods, positive matrix factorization (PMF), which limits factors to those with positive values, is one of the most actively used receptor models worldwide, including in the USA (Paatero and Tapper 1994; Polissar et al. 2001; Han et al. 2017), South Korea (Kim et al. 2018; Park et al. 2020), China (Zong et al. 2016; Wu et al. 2018; Zhao et al. 2019; Lv et al. 2021), and Vietnam (Cohen et al. 2010). PMF modeling has its own error review capabilities, such as bootstrapping (BS) and displacement (DISP), which leads to a relatively accurate source apportionment and is useful for interpreting source profiles based on domain knowledge. In addition, new approaches have been attempted to improve their usability (Brown et al. 2015; Wang et al. 2018; Du et al. 2021). More recently, advanced methods such as dispersion normalized (DN) PMF have emerged (Dai et al. 2020, 2021), and matrix factorization with Bayesian methodology has also been used in receptor models (Park and Oh 2015; Park et al. 2018, 2021).

The health risk assessment coupled with source apportionment can be used to develop more specific environmental health policies because the health risks due to exposure to PM_2.5_ may vary depending on the emission source (Yang et al. 2013; Leogrande et al. 2019; Kim et al. 2019; Wang et al. 2020; Zhang et al. 2020). It is shown that oxidative potentials per PM mass differs greatly depending on the emission sources such as vehicle exhaust and secondary aerosols (Shiraiwa et al. 2017). Accordingly, health risk assessments by sources were considered essential for comprehensive understanding behavior of particulate matter (PM) (Li et al. 2013; Fan et al. 2021; Choi et al. 2022). Also, although the importance of evaluation of ambient PM that takes into consideration size, chemical composition, and source of particles has been pointed out (Cassee et al. 2013), those factors have rarely been involved in the health or toxicity assessment (Hannigan et al. 2005; Kim et al. 2020; Fushimi et al. 2021). Recent relevant studies have investigated specific sources and chemical components of air pollution that affect human health and compared the assessment results to those of other regions, but these studies are still lacking (Fan et al. 2021).

To date, far too little attention has been paid to conduct both source apportionment and health risk assessment simultaneously in middle-sized industrial cities that could exist in any country in the world, and rather, only some large cities are being studied (Hu et al. 2012; Yang et al. 2013; Fu et al. 2021). Air pollution is generally more severe in industrial areas, owing to local industrial emissions (Fu et al. 2021; Shende and Qureshi 2022). The negative impact to human health in these areas are expected to be greater than those to humans in areas with less pollution because of the presence of pollutants such as heavy metals, organic carbon (OC), or elemental carbon (EC) (Samara et al. 2003; Kumar et al. 2020). Therefore, the method source apportionment integrated with health risk assessment needs to be applied as a basis for the development of air pollution management policies, especially in industrial areas.

The main purpose of this study was to identify the sources of PM_2.5_ and to evaluate the health risk of each source type in Siheung, which is a city with national industrial complexes located in the Republic of Korea. The specific aims of this study were to (1) identify and apportion PM_2.5_ sources with error estimation, (2) assess health risks of PM_2.5_ inhalation and the contribution of each source to these health risks, and (3) identify the characteristics of the sources that represent higher health risks and explore appropriate PM_2.5_ reduction measures based on a source-based health risk assessment. The target area of this study is a medium-sized industrial city, which is similar to many other industrial cities worldwide.

## Materials and methods

### Study site, sampling, and analysis

**Fig. 1:**
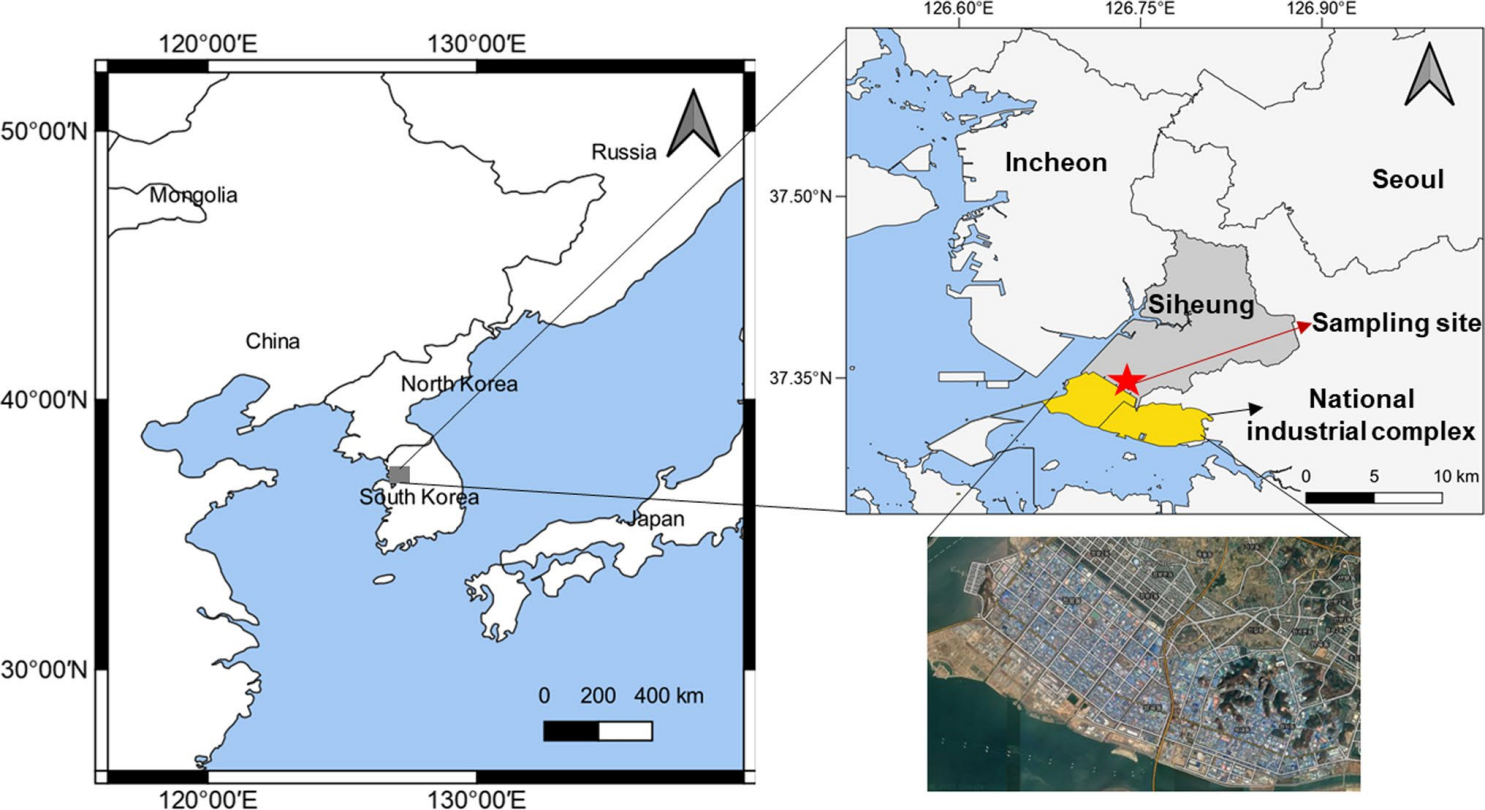
Locations of this study site (Siheung city and sampling site)

**Fig. 2:**
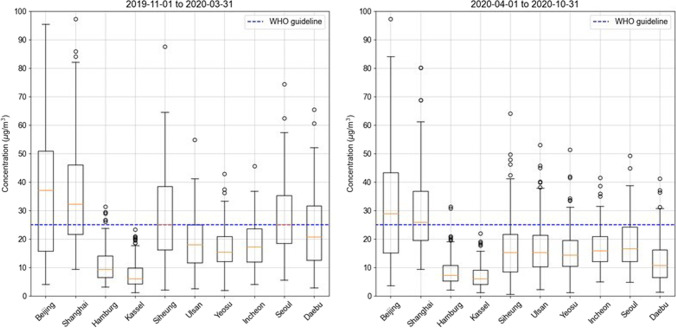
Average daily PM_2.5_ concentration comparisons between the sampling site and other sites

Siheung City is located at approximately 20 km southwest of Seoul, Republic of Korea, and it has a population of approximately 0.56 million (as of 2021). In the southwest of Siheung City, 10,000 factories are located in a national industrial complex, with an area of approximately 165 million m^2^ (Siheung City’s official website, https://www.siheung.go.kr/english/, last access: 10 August 2021). The main industrial fields include textiles, chemicals, metal smelting, printing, and paper. Siheung City has high accessibility to Seoul owing to the highways and nearby ports; therefore, industrial activities are prominent in that area. It shares city-regional characteristics with medium-sized industrial cities in other major countries worldwide. Figure 1 illustrates the location of Siheung City and its industrial complexes. The daily average PM_2.5_ concentrations in Siheung City were compared with those of other industrial cities in Korea, China, and Germany. Figure 2 shows the PM_2.5_ concentration levels of industrial cities in China and Germany (Beijing, Shanghai, Hamburg, Kassel), in Korea (Ulsan, Yeosu, Incheon, and Daebudo), and Seoul, the capital city of Korea. For the data, the air quality index value obtained from the Air Quality Historical Data Platform (https://aqicn.org/, last access: 10 August 2021) was converted into mass concentration.

To quantify the chemical composition of PM_2.5_, samples were collected every three or four times a week over 24 h from November 2019 to December 2020 at the rooftop of Jeongwnag-dong National Air Quality Measuring Station (37.3472°N, 126.7399°E, shown as a red star in Fig. 1), which is approximately 10 m above the ground level. A PM_2.5_ sampler (PMS-204, APM Engineering, South Korea) with three parallel channels was used to collect PM_2.5_ samples. Two channels were installed with Teflon filters (2 μm pore size and 47 mm diameter, Measurement Technology Laboratories, USA) and one channel with a quartz filter (47 mm diameter, Pall Corporation, USA). Each sampler was operated for 24 h at a 16.67 L/min flow rate. The mass concentration, ionic component, OC, EC, and elemental components of PM_2.5_ were analyzed as follows. The mass concentration was calculated by measuring the weight of a 24 h dried Teflon filter (PT47P, MTL, US) before and after sample collection, and then dividing the obtained value by the collected air volume. The weight of the filters was measured after removing static electricity at a constant temperature (21 ± 1.5 °C) and humidity (35 ± 5%). Moreover, the weight of the blank filter was measured and used for correction. Ion component analysis was performed by ion chromatography (930 Compact IC Flex, Metrohm, Switzerland) using a Teflon filter (TF-10000, PALL, USA). In the analysis, each of the entire sampled filter was extracted for 120 min in a bath-type sonicator using 40 ml of distilled water, and then filtered using a 0.45 µm membrane. For OC and EC, a quartz fiber filter paper (7407, PALL, USA) cut to a diameter of 4 mm in the sampled portion was used, and the analysis was performed using the thermal optical transmittance (TOT) method in a carbon analyzer (laboratory OC-EC aerosol analyzer, Sunset Lab, USA), and the analysis conditions followed the NIOSH 5040 protocol. The trace elements were analyzed by energy dispersive X-ray fluorescence (ED-XRF) spectroscopy (ARL QUANT'X ED XRF Spectrometer, Thermo Fisher Scientific, USA) using Teflon filters (PT47P, MTL, US) without additional pretreatment. Namely, each of the entire sampled filter was used in the measurement. A total of 29 components were analyzed. Including the mass concentration analysis, 6 ionic species (NO_3_^−^, SO_4_^2−^, NH_4_^+^, K^+^, Na^+^, and Cl^−^), carbons (OC and EC), and 21 species of elemental components (Na, Mg, Al, Si, S, Cl, K, Ca, Ti, V, Cr, Mn, Ba, Fe, Ni, Cu, Zn, As, Se, Br, and Pb) were quantified.

### Positive matrix factorization modeling and combined analysis with meteorological data

The positive matrix factorization (PMF) model has been widely used as a method of factor analysis to derive air pollution sources from speciated sample data (Paatero and Tapper 1994; Paatero 1997; Hopke 2016). The data matrix can be separated into factor contributions (G) and factor profiles (F) (United States Environmental Protection Agency (US EPA 2014). The equation for the PMF model is given by Paatero and Tapper (1994).

1 $$\mathrm{X}=\mathrm{G}\times \mathrm{F}+\mathrm{E}$$

where X is a matrix of the sample dataset (e.g., *n* $\times$ j matrix, where *n* is the sampled date and *j* is the chemical species of the data), G is the source contribution matrix (e.g., *n* $\times$ *q* matrix, where *q* is the source contribution), F is the source profile matrix (e.g., *q* $\times$ *j* matrix), and E is a residual matrix (e.g., *n* $\times$ *j* matrix).

In Eq. (1), all elements of matrices G and F are constrained to positive values. To derive the appropriate G and F matrices, the objective function Q in Eq. (2) was minimized (Paatero 1997).

2 $$\mathrm{Q}= \sum_{i=1}^{n}\sum_{j=1}^{m}{\left(\frac{{e}_{ij}}{{\sigma }_{ij}}\right)}^{2}$$

where *n* is the number of samples, *m* is the number of species, ${e}_{ij}$ is the residual (e.g., element of matrix E), and ${\sigma }_{ij}$ is the data uncertainty (e.g., uncertainty of chemical species *j* at date *i*).

The US EPA PMF version 5.0.14 was used to estimate the source contribution and profile in the target area. The concentration data for the modeling included the pre-processed chemical composition analysis of 22 substances (NO_3_^−^, SO_4_^2−^, NH_4_^+^, K^+^, Na^+^, Cl^−^, OC, EC, Mg, Al, Si, Ca, Ti, V, Cr, Mn, Fe, Ni, Cu, Zn, As, and Pb) and PM_2.5_ mass concentration. The pretreatment process considered the ratio of cations and anions in PM_2.5_, and data were excluded if concentrations were below the detection limit or when an outlier was detected. If there were duplicate measurements, one was selected for use. Data with an S/N ratio of 0.2 or less were also removed. This method is an established procedure reported in previous studies (Choi et al. 2013; Kim et al. 2018; Park et al. 2020). The data uncertainty was calculated using Eq. (3), according to the US EPA guidelines (US-EPA 2014).

3 $$\sigma_{ij}=\left\{\begin{array}{c}\left(5/6\right)\times MDL\\\sqrt{\left(Conc.\times0.1\right)^2+\left(0.5\times\mathrm{MDL}\right)^2}\end{array}\right.\begin{array}{c}\left(\mathrm{if}\;\mathrm{Conc}.\leq\mathrm{MDL}\right)\\\left(\mathrm{if}\;\mathrm{Conc}.>\mathrm{MDL}\right)\end{array}$$

where MDL is the method detection limit and Conc. is the concentration (μg/m^3^) of the species, (e.g., X_ij_). MDL values of the elemental components are listed in Table S1.

The data used for the modeling included 95 daily average values. The number of sources (e.g., *q*) in the model was selected by repeated modeling. Moreover, BS and DISP analyses in the US EPA PMF 5.0 were conducted to confirm the appropriate range of major chemical species by source. These functions are widely used to investigate errors and rotational ambiguity (Dai et al. 2020). PMF results of 8 to 10 factors were considered for the best solution.

The CPF analysis was applied to investigate source directionality and the PSCF analysis was applied to locate possible source areas. The hybrid single-particle Lagrangian integrated trajectory (HYSPLIT 5) model and gridded meteorological data from the US National Oceanic and Atmospheric Administration were used to calculate air parcel backward trajectories.

The conditional probability function (CPF) enable to analyze the changes in PM_2.5_ concentrations for each source according to wind direction and speed (Carslaw 2015). The CPF is defined as CPF = m_θ_/n_θ_, where m_θ_ represents the samples above a certain concentration in the wind direction θ, and n_θ_ is the total numbers of samples in the same wind direction. CPF values were visualized using hourly wind direction and speed data combined with PMF source contributions using the OpenAir package in R (version 4.0.3, Vienna, Austria). Meteorological data were obtained from the weather station located at the same position as the sampling site (37°20′48′′N 126°44′24′′E) and operated by the Korea Meteorological Administration (data are available at https://data.kma.go.kr/, last access: 10 August 2021). The upper 25% of PMF source contributions was used as the threshold criteria.

Subsequently, backward trajectory analysis was conducted using the Hybrid Single-Particle Lagrangian Integrated Trajectory (HYSPLIT) model. The transboundary airmass transport pathways from the sampling site were predicted. According to the sampling date, 24 h and 72 h of back trajectories were analyzed in 1 h increments. The possible past routes were tracked using the Global Data Assimilation System (GDAS) 1-degree meteorological data. The HYSPLIT version 5.0 and PySPLIT, which is a Python-compatible package (Warner 2018), were used. The potential source contribution function (PSCF) was calculated based on the results of the backward trajectory analysis. The PSCF model indicates the conditional probability of air coming from an area (Ashbaugh et al. 1985) and is represented by Eq. (4).

4 $$\mathrm{PSCF}= {m}_{ij}/{n}_{ij}$$

where ${m}_{ij}$ is the total number of trajectory endpoints that exceed the threshold concentration in the *i*, *j*th grid cell; and ${n}_{ij}$ is the total number of trajectory endpoints that pass the *i*, *j*th grid cell. In this study, the threshold concentration for ${m}_{ij}$ was in the 70th percentile.

The weighted PSCF (WPSCF) value can lead to more reliable results because the PSCF value can have high uncertainty in some cases (Polissar et al. 2001). Therefore, the WPSCF was calculated using Eq. (5). In addition, visualization was performed using WPSCF $\left({n}_{ij}\right)$ at each grid and interpolated by Kriging. The results and discussion of the combined analysis with meteorological data is provided in Text S1.

5 $$\mathrm{WPSCF}\left({\mathrm{n}}_{\mathrm{ij}}\right)=\left\{\begin{array}{c}1.0\times PSCF\left({\mathrm{n}}_{\mathrm{ij}}\right) ({\mathrm{n}}_{\mathrm{ij}}>3{\mathrm{n}}_{\mathrm{avg}})\\ 0.7\times PSCF\left({\mathrm{n}}_{\mathrm{ij}}\right) (3{\mathrm{n}}_{\mathrm{avg}}>{\mathrm{n}}_{\mathrm{ij}}>1.5{\mathrm{n}}_{\mathrm{avg}})\\ \begin{array}{c}0.4\times \mathrm{PSCF}\left({\mathrm{n}}_{\mathrm{ij}}\right) (1.5{\mathrm{n}}_{\mathrm{avg}}>{\mathrm{n}}_{\mathrm{ij}}>{\mathrm{n}}_{\mathrm{avg}})\\ 0.2\times \mathrm{PSCF}\left({\mathrm{n}}_{\mathrm{ij}}\right) ({\mathrm{n}}_{\mathrm{avg}}>{\mathrm{n}}_{\mathrm{ij}})\end{array}\end{array}\right.$$

### Health risk assessment

Using the species concentration for each source obtained through PMF modeling, the health risk was calculated following the guidelines established by the US EPA (2013, 2009). We evaluated only the substances with toxicity values, similar to previous studies on health risks of air pollution (Choi et al. 2011; Hu et al. 2012; Yang et al. 2013; Zhao et al. 2021; Fu et al. 2021). Therefore, the health risk results of this study did not reflect the ion components, OC, EC, and PM_2.5_ itself. The health risk was assessed only for toxic elements in PM_2.5_.

As inhalation is the predominant pathway for human exposure to PM_2.5_-bound toxic elements, we considered only the inhalation pathway for carcinogenic (As, Cr, Ni, and Pb) and non-carcinogenic (As, Cr, Cu, Ni, Pb, V, and Mn) risk estimations. For Cr, because its hexavalent and trivalent forms generate different levels of health impacts, the ratio of hexavalent to trivalent was set to 3:7 by referring to the abundance ratio in the PM of other industrial cities (Torkmahalleh et al. 2013; Widziewicz et al. 2016).

The average daily dose of PM_2.5_-bound trace elements via inhalation (ADD_inh_) was calculated using Eq. (6) (US EPA 2009).

6 $${\mathrm{ADD}}_{\mathrm{inh}} (\mathrm{\mu g}/{\mathrm{m}}^{3})=\frac{\mathrm{C}\times \mathrm{ET}\times \mathrm{EF}\times \mathrm{ED}}{\mathrm{AT}}$$

where C represents the mean concentration of a pollutant in the air (μg/m^3^) over the sampling period, and ET is the exposure time (h/d). EF is the frequency of exposure (365 days/year), ED is the exposure duration (y), and AT is the average time in h ($\mathrm{ED}\times 365\times$ 24).

The health risk assessment was based on adults residing in Korea. The exposure parameters used in the cancer and non-cancer risk assessments and their sources are listed in Table S2.

To estimate the carcinogenic risk by inhalation of PM_2.5_-bound trace elements, the incremental lifetime cancer risk (ILCR) was calculated following the risk assessment guidelines established by the US EPA (2009, 2013). The ILCR_inh_ was calculated using Eq. (7) (US EPA 2009).

7 $$\mathrm{ILCR}_{\mathrm{inh}}=\mathrm{ ADD}_{\mathrm{inh}}\times \mathrm{ IUR}$$

where IUR is the inhalation unit risk (m^3^/μg).

According to the US EPA(1998, 2013), an ILCR lower than 1 × 10^−6^ is regarded as negligible, an ILCR above 1 × 10^−4^ is likely to be harmful to human beings, and an ILCR value between 1 × 10^−6^ and 1 × 10^−4^ indicates a tolerable risks, but needing risk reduction plans. The IUR values were based on credible values from the US EPA’s Integrated Risk Information System (IRIS), and the Office of Environmental Health Hazard Assessment, (OEHHA) from the US EPA (2021), depending on the element. Table 1 shows the IUR values of each element, their sources, and the calculation results of health effects.

**Table 1:** Toxicological data and carcinogenic risk of PM_2.5_ in Siheung

Chemical	IUR (m^3^/μg)	Critical effects*	Source**	ILCR	
				Using median concentrations	Using 95 percentile concentrations
As	4.3.E-03	Lung irritation, decreased production of both red blood cells and white cells, deoxyribonucleic acid (DNA) damage	IRIS	4.47E-06	1.17E-05
Cr^6+^	1.2.E-02	Liver and kidney disease, lung cancer	IRIS	2.04E-06	4.17E-06
Ni	2.4.E-04	Lung embolisms, lung and nasal cancer	IRIS	7.07E-08	1.30E-07
Pb	1.2.E-05	Renal impairment, encephalopathic signs	OEHHA	6.92E-08	1.72E-07

^*^ Critical effects indicated the major carcinogenic effects on humans listed in the literature (Briffa et al. 2020)

^**^ The sources listed were the original reference of the value, and the values were downloaded from US-EPA (https://www.epa.gov/risk/regional-screening-levels-rsls-generic-tables, last access: 10 August 2021)

The calculation method of non-carcinogenic risk is given in Text S1 and the results and discussion for the non-carcinogenic risk is provided in Text S2.

The health risks calculated in Siheung were compared to those in Seoul and Daebudo, of which measured data were obtained from the literature (Kim et al. 2018; Park et al. 2019). Median values and the same exposure parameters were used in the health risk estimation for the comparison using consistent manners. The period of available data was 2013–2014 for Seoul, 2019–2020 for Siheung, and 2016 for Daebudo.

## Results and discussion

### PM_2.5_mass concentration and chemical speciation

The average mass concentration of PM_2.5_ over the sampling period (11/16/2019 to 10/02/2020) was 23.5 ± 13.9 μg/m^3^. A time series plot is shown in Fig.S1 to compare the PM_2.5_ concentration data obtained in this study and those provided from a national monitoring station (https://www.airkorea.or.kr/, last access: August 10, 2021). Both time series presented a similar trend, which confirmed the validity of our data acquisition. High concentrations (over the Korean daily standard of 25 μg/m^3^) were observed in 37 of the 95 samples, primarily in winter and spring (35 cases from November to May). The detailed concentrations of PM_2.5_ and chemical species (29 species) are summarized in Table S3.

The PM_2.5_ concentration levels in Siheung and other cities are shown in Fig. 2. The average daily PM_2.5_ concentration in Siheung was similar to that in Seoul and higher than those in Yeosu and Ulsan, which are industrial cities in South Korea. Seoul and Siheung are cities located in the northwest of South Korea and are known to be affected by long-range transport of PM_2.5_ from China (Bae et al. 2019; Kumar et al. 2021). The contribution of long-range transport from China to PM_2.5_ in Seoul was estimated ranged from 41 to 44% between 2012 and 2016 (Bae et al. 2019), approximately 20% in August, and approximately 60% in January and February (Kumar et al. 2021). In comparison to industrial cities of other countries, the average PM_2.5_ concentration in Siheung was higher than those in Hamburg and Kassel, in Germany, and lower than those in Beijing and Shanghai in China. This suggests that source apportionment coupled with health risk assessment in Siheung may be an example of a small and medium-sized industrial city with moderate PM_2.5_ pollution.

As the measurement and analysis period of this study included the COVID-19 lockdown or social distancing period in neighboring countries and Korea, we evaluated possible interferences. A previous study on air quality change in Seoul under COVID-19 social distancing reported that the monthly average PM_2.5_ concentration (from 29 February to 29 March 2020) decreased by 10.4% in 2020, which was contrary to the average increase of 23.7% over the corresponding periods in the previous 5 years (Han et al. 2020). Je et al. (2021) also reported that the mean PM_2.5_ level in 2020 decreased by 16.98 μg/m^3^ nationwide in Korea compared to 2019, which represented a decrease of 45.45% (*p* < 0.001). However, significant reductions in PM_2.5_ were observed in Korea even before social distancing owing to the changes in transboundary PM_2.5_ concentration (Kim and Lee 2018). In China, the average PM_2.5_ concentration during the lockdown period (January to February 2020) was 18 μg/m^3^, which represented a reduction of 30–60% in most regions (Bai et al. 2021).

Although there may be a gap between present results and previous ones, comparison with previous data is essential to obtain detailed information on PM_2.5_ pollution. A comparison of average concentrations of PM_2.5_-bound chemicals obtained in this study and those by Park et al. (2019) in Seoul indicated that Siheung had a higher concentration of Cr than Seoul. The average concentrations of As, Pb, Cr, Mn, Ni, Cu, Zn, and V, which are major toxic elements, were 4.74, 25.74, 2.43, 16.37, 1.26, 7.13, 73.55, and 0.40 ng/m^3^ in Sheung, and 5.53, 38.11, 1.74, 16.93, 2.11, 7.92, 100, and 4.30 ng/m^3^ in Seoul (Park et al. 2019) respectively. The concentrations of toxic elements except Cr were higher in Seoul than in Siheung. However, further research is required to determine the impacts of reduced concentrations attributed to the effects of the COVID-19. When comparing the concentrations of elements in Siheung and Seoul during the sampling period of this study, the mean concentrations of Pb, Cr, Mn, Ni, Cu, Zn, and V in Siheung were 1.6, 3.0, 2.2, 4.0, 2.8, 2.2, and 1.4 times higher than those in Seoul (Korea Ministry of Environment and National Institute of Environmental Research 2022), respectively. These results might indicate that Siheung has a high concentration of Cr and other elements because the concentrations were high even during the COVID-19 lockdown period. This was suggested because these elements are considered chemical markers of combustion and traffic sources (Farahani et al. 2021), which were reduced during the lockdown period. In Beijing, the mean concentrations of PM_2.5_-bounded As, Pb, Cr, Mn, Ni, Zn, and V during the winter of 2018 were 4, 44, 15, 34, 8, 110, and 7 ng/m^3^ (Fan et al. 2021), respectively, which are overall higher than those obtained in Siheung. The concentrations of the clean case presented in the literature showed similar results to those of Siheung. In Quebedo, Portugal (Silva et al. 2020), the concentrations of As, Cr, and Zn were 0.44, 3.55, and 11.0 ng/m^3^, which were lower than those in Siheung, Korea.

### Source apportionment of PM_2.5_ by PMF modeling

**Fig. 3:**
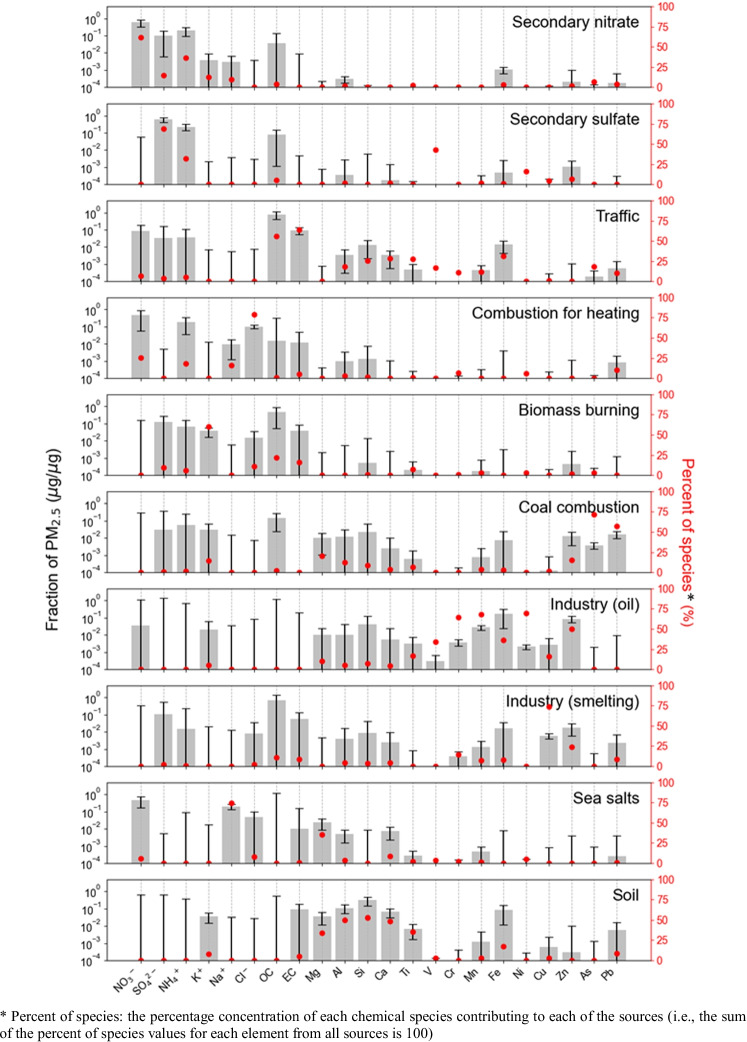
Source profile results of PMF modeling with DISP errors (The black bar corresponds to the left axis, and the red dot corresponds to the right axis)

**Fig. 4:**
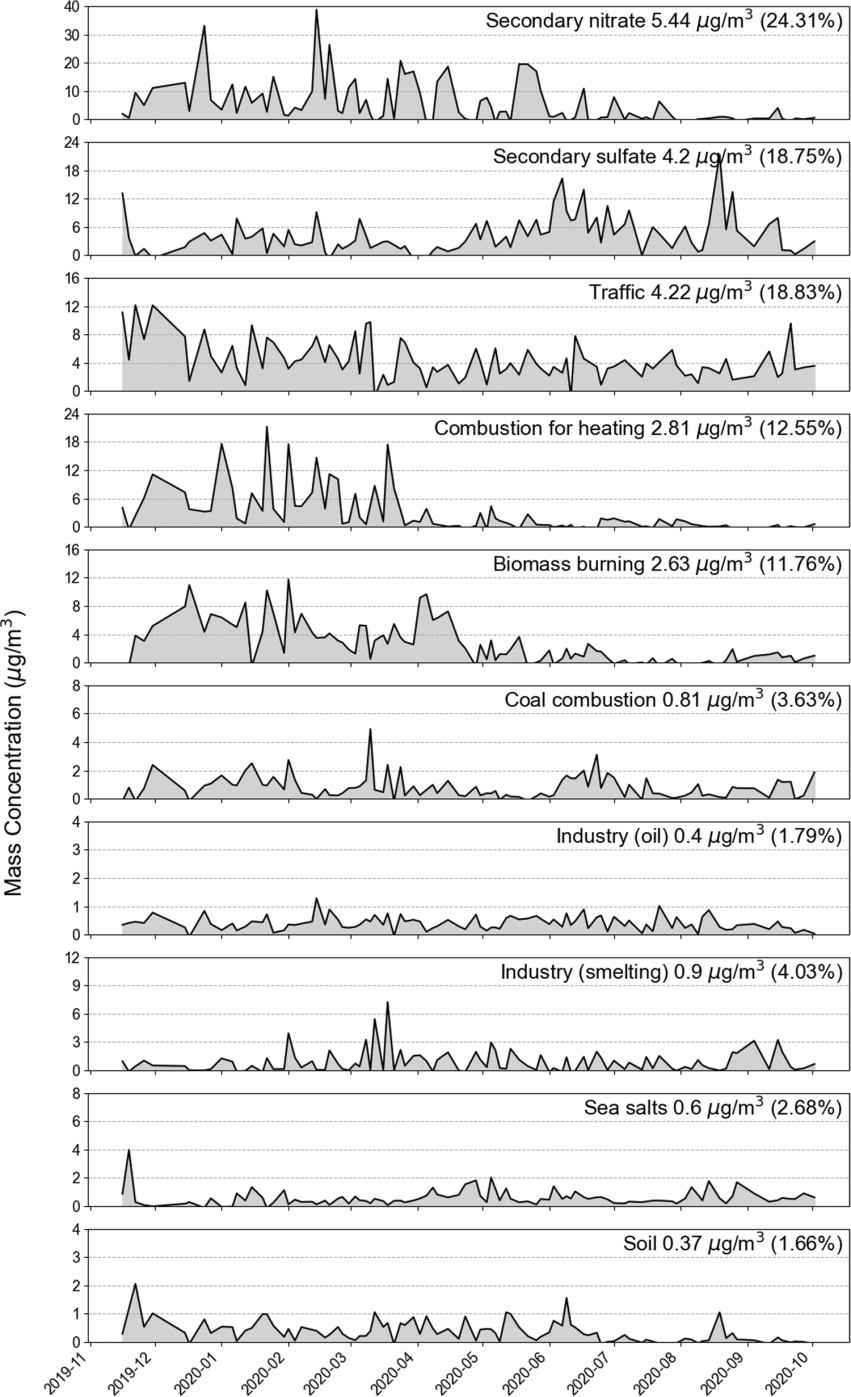
Source contribution time-series plot of PM2.5 in Siheung, Republic of Korea

The source profile and the time series of PMF factor contribution are shown in Fig. 3 and Fig. 4, respectively. Total 10 sources of PM_2.5_ were identified, and all major species of the sources were within the DISP intervals (Fig. 3). The R^2^ between observed and predicted PM_2.5_ concentrations for the best solution was 0.92, indicating a reasonable modeling result. The 10 sources included secondary nitrate, secondary sulfate, traffic, combustion for heating, biomass burning, coal combustion, heavy oil industry, smelting industry, sea salts, and soil. The sources with the highest contributions were the secondary-generated particles (secondary nitrate and sulfate) (Fig. 4).

Secondary nitrate had an average contribution of 24.3% to PM_2.5_ mass concentration. The concentration of secondary nitrate was relatively high in the winter when the temperature was low (Fig. 4). The main species of secondary nitrate are NH_4_^+^ and NO_3_^−^, which are formed in urban air primarily through gas-particle partitioning (Shi et al. 2019). This occurs because nitrogen oxide and ammonia gas, which are gaseous precursors in spring and winter, easily react in the atmosphere producing particulate nitrate (Choi et al. 2013; Park et al. 2020). Secondary sulfate (18.8%) was identified by the high concentrations of SO_4_^2−^ and NH_4_^+^ (Park et al. 2020). The contribution of secondary sulfate tended to increase primarily in the summer. This is considered to reflect the formation of sulfate in the atmosphere that becomes active when both temperature and humidity are high (Heo et al. 2009).

Traffic was identified as a source using OC and EC as major indicator components, and it contributed to 18.8% of the PM_2.5_. The high component ratio of carbon species exhibited the characteristics of automobile pollutants. Fe is also considered as an indicator of traffic resuspension as it is emitted from the brake wear of gasoline and diesel-powered engines (Belis et al. 2013).

Combustion for heating as a pollution source was characterized by the high Cl^−^ content (Tian et al. 2020), and it presented a high contribution from November 2019 to March 2020. This period coincided with the heating periods in Korea and northern China. The combustion for heating contributed to 12.6% of the PM_2.5_.

Biomass burning contributed to 11.8% of PM_2.5_, with K^+^ as its major component (Andreae 1983). Its contribution was identified by the high load of OC and the medium load of EC (Moon et al. 2008; Liu et al. 2017). In addition, biomass burning exhibited seasonal characteristics with a high contribution in the winter (Shi et al. 2014), which is consistent with the increase in the use of wood fire for domestic heating (Choi et al. 2013).

Coal combustion contributed to 3.6% of PM_2.5_, and As and Pb were considered its major indicator components. The contribution of coal combustion did not exhibit any distinct seasonal fluctuations, which was consistent with the characteristics of local sources. For example, Arsenic is known as a major marker of coal combustion pollution (Duan and Tan 2013), and it is known to be largely emitted from fossil fuel burning.

Industrial sources were divided into heavy oil- and smelting-related sources. The high ratio of V and Ni was considered a characteristic of heavy oil-based industrial sources (Jang et al. 2007). For industrial smelting sources, the major indicators were heavy metal components such as Cu, Cr, Mn, Pb, and Zn (Dai et al. 2015). The industrial contributions did not show significant seasonal fluctuations.

Sea salt sources were identified by high concentrations of Na, Mg, and K (Park et al. 2020). The source was referred to as a fresh seal salt because of the relatively high concentration of chlorine ions (Han et al. 2017). Its concentrations exhibited seasonal characteristics, and the highest contributions were observed during the winter. Finally, soil sources were identified by the existence of representative crustal components such as Mg, Al, Si, Ca, and Ti (Thorpe and Harrison 2008; Liu et al. 2017) and they contributed to 1.7% of PM_2.5_.

Park et al. (2020) performed PMF modeling in Seoul in 2014–2015 and isolated 9 sources. The contributions of secondary sources and traffic sources in Seoul were 6.3 and 5.3 µg/m^3^ higher than those in Siheung, respectively. Unlike in the study of Seoul (Park et al. 2020), the industrial smelting source was extracted in this study probably due to non-ferrous smelter sources in the near national industrial complex. The existence of a smelting source was also observed in a PMF modeling study in Daebudo (Kim et al. 2018), near Siheung. In the literature, Cu, Zn, and Pb have been designated as major markers of industrial smelting sources (Kim et al. 2018).

### Carcinogenic health risks

The uncertainty of health risk estimates coupled with PMF modeling results was calculated. The difference between the health risks using the measured values and the health risks coupled with PMF model results was within 10% (data not shown).

**Fig. 5:**
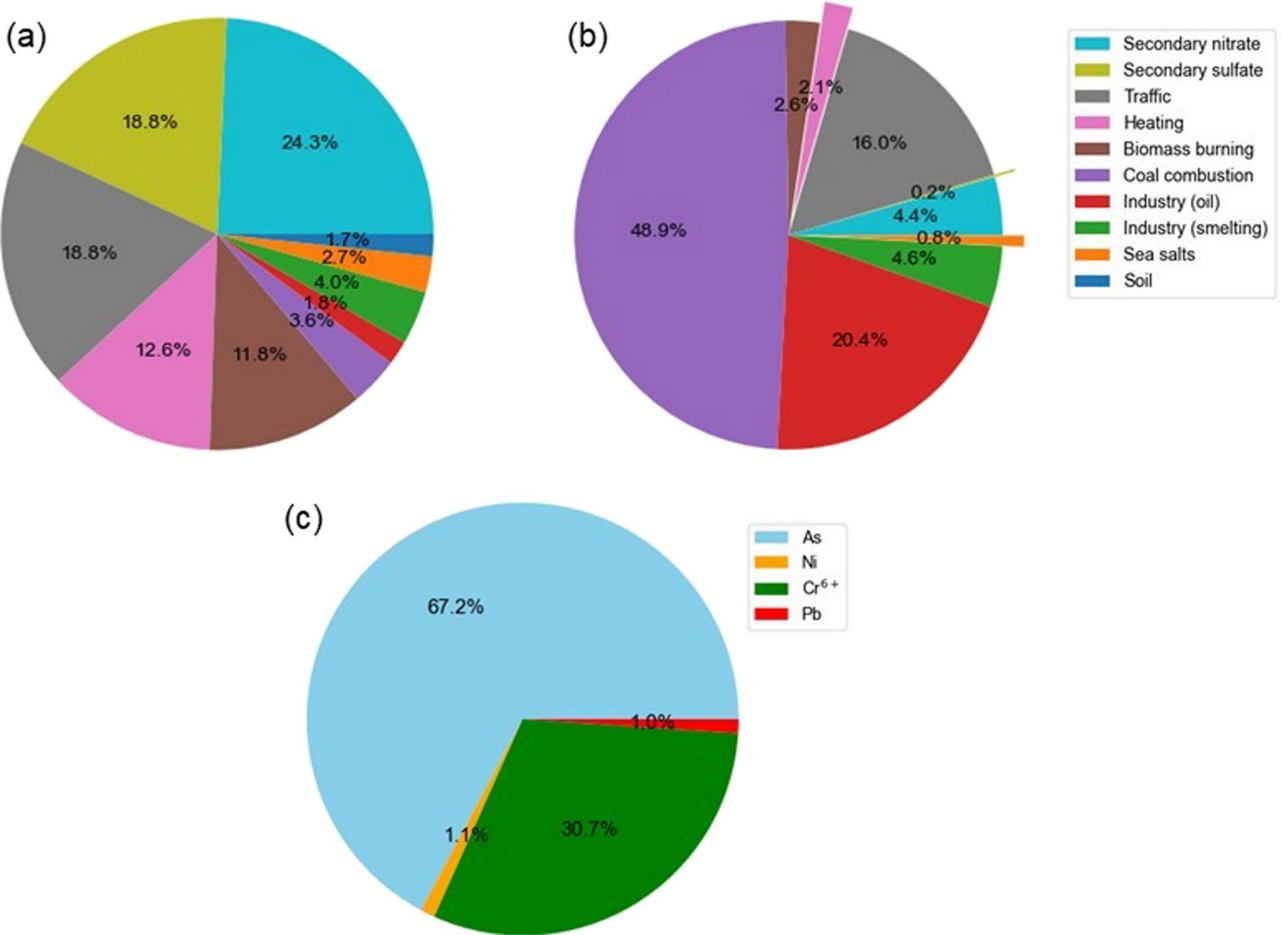
Annual average contributions **a** of sources to PM_2.5_ mass concentrations, **b** of sources to cancer risks, and **c** of elements to cancer risks

The calculated carcinogenic health risks by elements were shown in Table 1. The obtained carcinogenic health risks indicated that both the median and 95 percentile concentrations of As and Cr^6+^ exceeded the ILCR value of 1E-06, whereas the ILCR values of Ni and Pb did not exceed the reference value (Table 1). These results suggest that air pollution management in Siheung should be based on pollution sources, focusing on As and Cr sources. This can also be confirmed in Table 2, which presents the health risk assessment results by element and source. According to the estimated health risks from PM_2.5_ sources using the median concentrations, the sources with high health risk potentials were coal combustion, oil industries, and traffic, which accounted for 48.9%, 20.4%, and 16.0% of the total ILCR value, respectively (Table 2). The concentration of portioned As and Cr had the greatest influence on the health risk values of each source. However, the absolute contributions of them to PM_2.5_ mass concentrations were 3.6%, 1.8%, and 18.8%, respectively (Fig. 4). Figure 5 shows annual average contributions of sources to PM_2.5_ mass concentrations and to cumulative cancer risk, and of elements to cumulative cancer risks. The contributions of sources to PM_2.5_ mass concentration and to health risks were very different. Therefore, the contribution of PM_2.5_ sources might not be representative of health risks, which supports the argument that to manage PM_2.5_ with a focus on health risks, the concentration of toxic metal elements should be considered rather than total mass concentration. (Farahani et al. 2021).

**Table 2:** Estimated carcinogenic risk in Sihueng (median elemental concentrations used)

Source	Toxic elements in PM_2.5_				Sum of incremental cancer risk by source
	**As**	**Cr**^**6+**^	**Ni**	**Pb**	
Secondary nitrate	2.90E-07	–	–	2.86E-09	2.93E-07 (4.4%)
Secondary sulfate	–	–	1.14E-08	–	1.14E-08 (0.2%)
Mobile	8.34E-07	2.30E-07	–	7.07E-09	1.07E-06 (16.0%)
Combustion for heating	–	1.32E-07	4.51E-09	7.17E-09	1.44E-07 (2.1%)
Biomass burning	1.52E-07	2.12E-08	2.19E-09	–	1.75E-07 (2.6%)
Coal combustion	3.24E-06	–	–	4.02E-08	3.28E-06 (48.9%)
Industry (oil)	–	1.32E-06	4.93E-08	–	1.37E-06 (20.4%)
Industry (smelting)	–	3.02E-07	–	6.26E-09	3.08E-07 (4.6%)
Sea salts	–	5.11E-08	3.53E-09	4.61E-10	5.51E-08 (0.8%)
Soil	–	–	2.60E-10	6.13E-09	6.39E-09 (0.1%)
**Sum of incremental cancer risk by element**	4.52E-06 (67.2%)	2.06E-06 (30.7%)	7.12E-08 (1.1%)	7.02E-08 (1.0%)	6.71E-06 (100%)

The concentrations of As and Cr that must be reduced to achieve negligible health effects were calculated. The results indicate that to reduce the health risks of As to below 1E-06, the As concentration should be reduced to 1 ng/m^3^ or less, which represents a reduction of at least 75% compared to the current level. For Cr, the required concentration reduction was at least 50%. Therefore, there is a need for a significant reduction in coal combustion, which is the main source of As pollution, and in emissions from the oil industry, which are the main sources of Cr. In addition, as the seasonal differences in ILCR were not significant (data not shown), an overall reduction is necessary, instead of a specific-season reduction plan.

Strengthening the control of pollutants emitted from industrial sources is an important environmental and public health issue. Therefore, the industrial emission sources of As and Cr in cities such as Siheung need to be managed, and efforts to reduce ambient concentrations need to be taken. Owing to the COVID-19 pandemic, industrial activity and traffic were likely restricted compared to usual rates during this study. This is supported by Dai et al. (2021), who reported that human activities, such as industry and transportation, declined during the epidemic outbreak and spread. Therefore, it is possible that the health risks assessed in this study were underestimated. Therefore, further studies beyond the pandemic period are needed for an accurate estimation of health risks.

The calculated ILCR values for Siheung (2019–2020), Seoul (2013–2014), and Daebudo (2016) are shown in Table S4. The results of Seoul were calculated from the data of Park et al. (2019), and the results of Daebudo were calculated from the data of Kim et al. (2018). The health risk from As in Siheung (4.52E-06) was lower than those in Seoul (1.35E-05) and Daebudo (3.02E-06). This result might have been obtained because the Siheung data reflected an underestimation of the decrease in human activity owing to the COVID-19 pandemic. The health risk values in Nanjing (Hu et al. 2012) and Beijing (Fan et al. 2021) in China were 9.04E-06 and 1.67E-06, respectively, which were similar to the value in Siheung. These results indicate that As presents a health risk even at low concentrations (ng/m^3^). This is consistent with previous studies suggesting that the presence of As in the atmosphere is a major public concern for human health (Widziewicz et al. 2016). Nevertheless, the health risk of Cr^6+^, Siheung, and Seoul also exceeded 1E-06, and Siheung presented the highest value (2.06E-06); therefore, Cr pollution in Siheung should be carefully managed. A similar observation of Cr-dominated carcinogenic risk from industrial and traffic sources has been reported in Delhi, India (Khillare and Sarkar 2012). Hu et al. (2012) and Fan et al. (2021) reported that the carcinogenic risks of Cr for adults from PM_2.5_ in Nanjing and Beijing were 8.70E-05; and 2.2E-05, respectively, which are approximately 20.9 and 5.3 times the value in Siheung. The industries were identified as Cr sources in this study (Fig. 3). Accordingly, Fan et al. (2021) identified the metal smelting industry as the main source of Cr.

## Conclusion

Ten types of PM_2.5_ emission sources were derived using a PMF model in Siheung, South Korea. Based on the sources derived, the carcinogenic and non-carcinogenic health risks due to PM_2.5_ inhalation were estimated. For coal combustion, heavy oil industry, and traffic sources, the contribution to PM_2.5_ mass concentration was low but exceeded the benchmark carcinogenic health risk value (1E-06). Therefore, countermeasures on the PM_2.5_ emission sources are better to be performed not only based on the PM_2.5_ mass concentration but also based on the health risks. In order to manage the effects of PM_2.5_ on human health in industrial cities, it is necessary to reduce the concentration of major toxic elements (especially As and Cr) and manage the emission sources. The methodology used in this study, which combines PMF modeling and health impact assessment, can be used to derive source types and calculate health impacts by source in other cities.

## Supplementary Information

Below is the link to the electronic supplementary material.

Supplementary file1 (DOCX 1573 KB)

## Data Availability

All data generated or analyzed during this study are included in this published article and its supplementary information files. Also, Sources of publicly available internet data are indicated in the text.
