# Supplementary material for: Health risk assessment and source apportionment of PM2.5-bound toxic elements in the industrial city of Siheung, Korea
Source: Environ Sci Pollut Res Int. 2022 May 4;29(44):66591–604. doi: 10.1007/s11356-022-20462-0 (PMC9066139; doi:10.1007/s11356-022-20462-0)
Supplement: Supplementary file 1 — Supplementary file1 (DOCX 1573 KB) [file 11356_2022_20462_MOESM1_ESM.docx]

**Supplementary Material**

**Health risk assessment and source apportionment of PM_2.5_-bound toxic elements in the industrial city of Siheung, Korea**

Young Su Lee^a^, Young Kwon Kim^b, e^, Eunhwa Choi^c^, Hyeri Jo^a^, Hyeseung Hyun ^d^, Seung-Muk Yi^b^, Jae Young Kim^a, *^

^a^ *Department of Civil and Environmental Engineering, Seoul National University, 1 Gwanak-ro, Gwanak-gu, Seoul, Republic of Korea*

^b^ *Department of Environmental Health Sciences, Graduate School of Public Health, Seoul National University, 1 Gwanak-ro, Gwanak-gu, Seoul, Republic of Korea*

^c^ *Institute of Construction and Environmental Engineering, Seoul National University, Gwanak-ro, Gwanak-gu, Seoul, Republic of Korea*

^d^ *College of Environmental Design, University of California, Berkeley, Berkeley, CA, USA*

^e^ *Division of Policy Research, Green Technology Center, Seoul 04554, Republic of Korea*

^*^Corresponding author: Department of Civil and Environmental Engineering, Seoul National University, 1 Gwanak-ro, Gwanak-gu, Seoul, Republic of Korea. Tel.: +82 2 880 8364; fax: +82 2 873 2684.

*E-mail address:* jaeykim@snu.ac.kr.

**Text caption**

Text S1. Results and discussion: probable source areas or directions

Text S2. Materials and methods: the description of non-carcinogen health risk calculation

Text S3. Results and discussion: non-carcinogenic health risk

**Table caption**

Table S1. Method detection limit (MDL) values of the elemental components (unit: ng m^-3^)

Table S2. Exposure parameters and input variables used in health risk calculation

Table S3. PM_2.5_ species concentrations in Siheung, Korea during the entire sampling period (11/16/2019 to 10/2/2020)

Table S4. Estimated carcinogenic and non-carcinogenic risks in Siheung, Seoul, and Daebudo, Korea (median concentration of each element used)

Table S5. Toxicological data and non-carcinogenic risks in Siheung

Table S6. Estimated non-carcinogenic risks in Sihueng (median elemental concentrations used)

**Figure caption**

Fig. S1. PM_2.5_ mass concentration comparisons between the sampled filter and the nearest national monitoring station. (a): time-series plot, and (b) 1:1 plot

Fig. S2 The CPF results of (a) industry (oil), (b) industry (smelting), (c) traffic, and (d) coal combustion sources

Fig. S3. PSCF results of PM_2.5_ sources in Siheung, Republic of Korea, 24-hour back trajectory of (a) Industry (oil); (b) Traffic; (c) Coal combustion, 74-hour back trajectory of (d) Industry (oil); (e) Traffic; (f) Coal combustion

**Text S1. Results and discussion: probable source areas or directions**

The probable emission locations were estimated for coal combustion, industries, and traffic sources, which presented a relatively high carcinogenic risk in the health risk assessment. The CPF results are shown in Fig. S2, and the PSCF results calculated through 24-h and 72-h back trajectory HYSPLIT analysis are shown in Fig. S3.

The CPF results for industrial sources indicated that the contribution of oil industries increased when the southwest winds of less than 4 m/s, that of smelting industries increased with southeast winds of 6 m/s or more. The results of the back trajectory analysis showed that the contribution of industries was widely distributed in southwest areas, from the Shandong Peninsula of China to the Taiwan region. According to Kim et al. (2018), the CPF of non-ferrous smelter sources pointed to the southeast of Daebudo, which was consistent with CPF results for smelting industry sources in this study. There are 4,632 high-tech manufacturing companies such as metal processing and machinery located in the national industrial complex of Siheung (as of 2019, Korea Statistical geographic information service, <https://sgis.kostat.go.kr/>, last access: last access: 10 August 2021), and more than 240,000 people are working in related industries. PM emitted from such industrial complexes was presumed to be industry (smelting) sources.

Coal combustion presented the highest contribution for northwest winds of approximately 2**−**6 m/s in the CPF plot (Fig. S2). In the 72-h back trajectory analysis (Fig. S3), PSCF was distributed along the Chinese coast from the west coast of Korea to the southwest of Korea. The results suggested that coal combustion sources presented high emissions from internal sources. Coal-fired power plants, petrochemical complexes, and Incheon ports are located around Siheung, so it was assumed that the influence of various sources was mixed. However, it was difficult to identify the specific locations, as there were various influencing factors in the vicinity. Long-term studies are required.

The CPF of traffic source showed that the contribution increased with slow winds of 3 m/s or less (Fig. S2). Siheung City has much traffic because of its proximity to Seoul and Incheon ports and it is presumed that this trend was well-reflected. The wind direction pattern also showed a result that was generally consistent with the arrangement of highways around the target area. The probability of the western sea of Korea was also high in the back-trajectory analysis (Fig. S3).

**Text S2. Materials and methods: the description of non-carcinogen health risk calculation**

The hazard quotient (HQ) and hazard index (HI) indicating the non-carcinogenic risk from PM_2.5_ bound toxic elements were calculated using Eq. (S1) and Eq. (S2), respectively (US EPA 2009).

|  | HQ = ADD_inh_/(RfC_i_×1,000 μg/mg)  HI = ∑ HQ_i_ | Eq. (S1)  Eq. (S2) |
| --- | --- | --- |

where RfC_i_ is the inhalation reference concentration (mg/m^3^) and i is the target element.

HI is a cumulative metric for HQs for individual toxic elements and exposure pathway. An HI value > 1 indicates the presence of non-carcinogenic risk, whereas values ≤ 1 indicate a negligible non-carcinogenic effect. The RfC_i_ values were determined according to the OEHHA, IRIS, and additional references (Agency for Toxic Substances and Disease Registry, ATSDR; Michigan Department of Environmental Quality, MDEQ; California Environmental Protection Agency, CalEPA) from the US EPA (2021). Table S5 shows the RfC_i_ values and health effect results for each element.

**Text S3. Results and discussion: non-carcinogenic health risk**

The non-carcinogenic health risks of all elements were less than 0.1 for both average and 95 percentile concentrations. Moreover, the HI value was 0.55, which did not exceed 1, thereby indicating a negligible toxic risk for all elements (Table S5). The maximum HQ value was 0.18 for As when the 95 percentile concentration was used. The calculations using median concentrations indicated that the pollutants with high toxicity values were the oil industry, coal combustion, and traffic (Table S6), which accounted for 37.4%, 30.5%, and 12.2% of the total HQ value, respectively. In contrast, according to the absolute contributions to PM_2.5_ concentration, their contributions accounted for 1.8%, 3.6%, and 18.8%, respectively (Fig. 4). According to the HQ results, Seoul had a higher non-carcinogenic health risk (at 0.585, which did not exceed 1) than Siheung and Daebudo. This was consistent with the results of a similar study in China (Hu et al. 2012), in which the calculated HI was less than 1 for adults, so that the non-carcinogenic health risks were considered of relatively low importance.

Table S1. Method detection limit (MDL) values of the elemental components (unit: ng m^-3^)

| Al | Si | Ca | Ti | V | Cr | Mn | Fe | Ni | Cu | Zn | As | Pb |
| --- | --- | --- | --- | --- | --- | --- | --- | --- | --- | --- | --- | --- |
| 6.69 | 5.54 | 4.39 | 3.72 | 0.201 | 0.726 | 0.969 | 7.04 | 0.609 | 0.242 | 1.22 | 1.42 | 3.19 |

Table S2. Exposure parameters and input variables used in health risk calculation

| **Factors** | **Definition** | **Units** | **Value** | **Source** |
| --- | --- | --- | --- | --- |
| C | The concentration of the metal in Ambient air | µg/m^3^ | Median, 95 percentile values | This study |
| ET | Exposure Time | hours/day | 6 | Fan et al., 2021 |
| EF | Exposure Frequency | day/year | 350 | This study |
| ED | Exposure Duration | year | 63.7 | Korean average (NIER, 2019) |
| AT | Average time | hours | 558,012 |  |

Table S3. PM_2.5_ species concentrations in Siheung, Korea during the entire sampling period (11/16/2019 to 10/2/2020)

| Species | Arithmetic mean  (ng m^-3^) | 25^th^ percentile  (ng m^-3^) | Median  (ng m^-3^) | 75^th^ percentile  (ng m^-3^) | Maximum  (ng m^-3^) |
| --- | --- | --- | --- | --- | --- |
| PM_2.5_ | 23,500 | 13,500 | 20,600 | 31,200 | 74,800 |
| NO_3_^-^ | 5,160 | 993 | 2,590 | 7,740 | 27,200 |
| SO_4_^2-^ | 3,580 | 1,800 | 3,260 | 4,380 | 14,100 |
| NH_4_^+^ | 2,910 | 1,330 | 2,710 | 4,100 | 12,100 |
| K^+^ | 166 | 58.9 | 139 | 239 | 525 |
| Na^+^ | 165 | 104 | 144 | 188 | 604 |
| Cl^-^ | 366 | 59.2 | 168 | 477 | 2,490 |
| OC | 5,830 | 3,760 | 5,330 | 7,370 | 15,400 |
| EC | 649 | 406 | 561 | 826 | 1,908 |
| Na | 187 | 136 | 172 | 222 | 536 |
| Mg | 41.0 | 27.5 | 34.8 | 49.7 | 159 |
| Al | 84.1 | 44.7 | 72.0 | 113 | 265 |
| Si | 222 | 107 | 185 | 296 | 665 |
| S | 1,850 | 1,130 | 1,740 | 2,310 | 6,200 |
| Cl | 505 | 113 | 248 | 772 | 2,560 |
| K | 233 | 108 | 196 | 328 | 766 |
| Ca | 51.4 | 28.2 | 43.3 | 66.6 | 233 |
| Ti | 7.41 | 4.38 | 6.37 | 9.93 | 20.1 |
| V | 0.396 | 0.196 | 0.319 | 0.531 | 1.41 |
| Cr | 2.43 | 1.21 | 2.25 | 3.14 | 8.25 |
| Mn | 16.4 | 10.5 | 16.2 | 21.4 | 44.5 |
| Ba | 6.25 | 3.01 | 4.45 | 7.33 | 30.9 |
| Fe | 188 | 124 | 171 | 239 | 458 |
| Ni | 1.26 | 0.788 | 1.14 | 1.65 | 3.38 |
| Cu | 7.13 | 1.98 | 4.77 | 10.3 | 45.0 |
| Zn | 73.5 | 42.4 | 60.6 | 98.8 | 226 |
| As | 4.74 | 1.90 | 3.34 | 6.61 | 27.3 |
| Se | 1.63 | 0.881 | 1.56 | 2.22 | 3.82 |
| Br | 13.6 | 5.99 | 9.78 | 14.9 | 168 |
| Pb | 25.7 | 11.8 | 21.3 | 31.6 | 111 |

Table S4. Estimated carcinogenic and non-carcinogenic risks of PM_2.5_ in Siheung, Seoul, and Daebudo, Korea (median concentration of each element used)

| **Toxic elements in PM_2.5_** | **Siheung, Korea**  **(2019.11 – 2020. 10)** | | **Seoul, Korea***  **(2013 – 2014)** | | **Daebudo, Korea****  **(2016)** | |
| --- | --- | --- | --- | --- | --- | --- |
|  | **ILCR** | **HQ** | **ILCR** | **HQ** | **ILCR** | **HQ** |
| As | 4.52E-06 | 7.01E-02 | 5.70E-06 | 8.84E-02 | 2.89E-06 | 4.47E-02 |
| Cr^6+^ | 2.06E-06 | 3.42E-02 | 1.50E-06 | 2.50E-02 | 8.63E-08 | 1.44E-03 |
| Cr^3+^ | - | 3.99E-03 | - | 2.92E-03 | - | 1.68E-04 |
| Cu | - | 8.63E-04 | - | 9.49E-04 | - | 1.07E-03 |
| Ni | 7.12E-08 | 2.12E-02 | 1.21E-07 | 3.61E-02 | 5.75E-09 | 1.71E-03 |
| Pb | 7.02E-08 | 3.90E-02 | 1.10E-07 | 6.09E-02 | 4.43E-08 | 2.46E-02 |
| V | - | 8.74E-04 | - | 1.03E-02 | - | 2.73E-02 |
| Mn | - | 7.84E-02 | - | 8.12E-02 | - | 3.84E-02 |
| **Sum** | **6.71E-06** | **2.49E-01** | **1.35E-05** | **5.85E-01** | **3.02E-06** | **1.39E-01** |

* Data source: (Park et al. 2019)

** Data source: (Kim et al. 2018)

**Table S5. Toxicological data and non-carcinogenic risk in PM_2.5_ of Siheung**

| Chemical | RfC_i_ (mg/m^3^) | Critical effects* | Source** | HQ | |
| --- | --- | --- | --- | --- | --- |
|  |  |  |  | Using median concentrations | Using 95 percentile concentrations |
| As | 1.5.E-05 | Heart problems, brain damage | OEHHA | 6.9E-02 | 1.8.E-01 |
| Cr^6+^ | 5.00E-06 | Allergic contact dermatitis and eczema, gingivitis | IRIS | 3.4E-02 | 7.0.E-02 |
| Cr^3+^ | 1.0.E-04 | DNA lesions (rarely toxic compared to hexavalent form) | ATSDR,2012 | 4.0E-03 | 8.1.E-03 |
| Cu | 2.0.E-03 | Insomnia, anxiety, restlessness | MDEQ, 2009*** | 8.5E-04 | 2.4.E-03 |
| Ni | 1.4E-05 | Asthma, allergic reactions, heart disorders | CalEPA | 2.1E-02 | 3.9.E-02 |
| Pb | 1.5.E-04 | Hypertension, miscarriages, stillbirth | IRIS | 3.8E-02 | 9.6.E-02 |
| V | 1.0.E-04 | Throat pain, headaches, impairment to the nervous system | ATSDR | 8.7E-04 | 1.7.E-03 |
| Mn | 5.00E-05 | Hypotension, pneumonia, sperm damage | IRIS | 7.8E-02 | 1.5.E-01 |
| HI (Summation) | | | | 0.25 | 0.55 |

* Critical effects indicated the major non-carcinogenic effects on humans listed in the literature (Briffa et al. 2020)

** The sources listed were the original reference of the value, and the values were downloaded from US-EPA (<https://www.epa.gov/risk/regional-screening-levels-rsls-generic-tables>, last access: 10 August 2021)

*** The value from MDEQ was accessed in the chemical update worksheet of the State of Michigan website (<https://www.michigan.gov/documents/deq/deq-rrd-chem-CopperDatasheet_527899_7.pdf>, last access: last access: 10 August

**Table S6. Estimated non-carcinogenic risk in Siheung (median elemental concentrations used)**

| **Source** | **Toxic elements in PM_2.5_** | | | | | | | | **Sum of incremental cancer risk by source** |
| --- | --- | --- | --- | --- | --- | --- | --- | --- | --- |
|  | **As** | **Cr^6+^** | **Cr^3+^** | **Cu** | **Ni** | **Pb** | **V** | **Mn** |  |
| Secondary nitrate | 4.50E-03 | - | - | - | - | 1.59E-03 | - | - | **6.1E-03 (2.5%)** |
| Secondary sulfate | - | - | - | 4.16E-05 | 3.39E-03 | - | 3.75E-04 | 1.08E-03 | **4.9E-03 (2.0%)** |
| Mobile | 1.29E-02 | 3.84E-03 | 4.47E-04 | 9.91E-06 | - | 3.93E-03 | 1.44E-04 | 9.14E-03 | **3.0E-02 (12.2%)** |
| Combustion for heating | - | 2.20E-03 | 2.56E-04 | - | 1.34E-03 | 3.98E-03 | 1.14E-06 | - | **7.8E-03 (3.1%)** |
| Biomass burning | 2.35E-03 | 3.54E-04 | 4.13E-05 | - | 6.51E-04 | - | - | 2.35E-03 | **5.7E-03 (2.3%)** |
| Coal combustion | 5.03E-02 | - | - | 1.29E-05 | - | 2.23E-02 | - | 3.07E-03 | **7.6E-02 (30.5%)** |
| Industry (oil) | - | 2.19E-02 | 2.56E-03 | 1.35E-04 | 1.47E-02 | - | 2.96E-04 | 5.35E-02 | **9.3E-02 (37.4%)** |
| Industry (smelting) | - | 5.03E-03 | 5.87E-04 | 6.36E-04 | - | 3.48E-03 | - | 5.68E-03 | **1.5E-02 (6.2%)** |
| Sea salts | - | 8.52E-04 | 9.94E-05 | 6.70E-07 | 1.05E-03 | 2.56E-04 | 3.06E-05 | 1.40E-03 | **3.7E-03 (1.5%)** |
| Soil | - | - | - | 2.70E-05 | 7.74E-05 | 3.40E-03 | 2.73E-05 | 2.24E-03 | **5.8E-03 (2.3%)** |
| **Sum of incremental cancer risk by element** | **7.01E-02 (28.2%)** | **3.42E-02 (13.8%)** | **3.99E-03 (1.6%)** | **8.63E-04 (0.4%)** | **2.12E-02 (8.5%)** | **3.90E-02 (15.7%)** | **8.74E-04**  **(0.4%)** | **7.84E-02 (31.6%)** | **0.25 (100%)** |


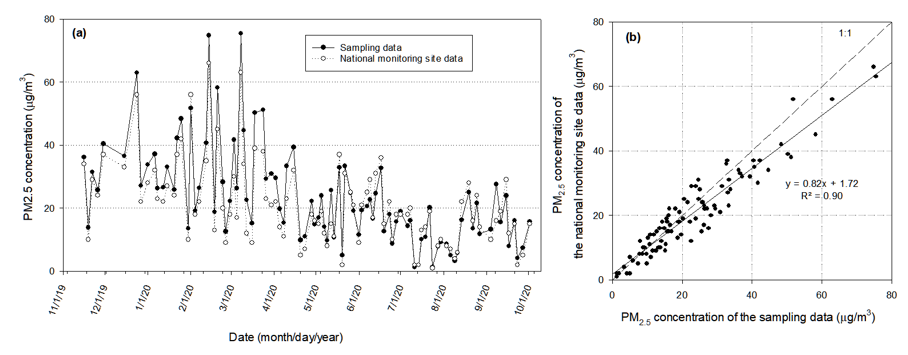


**Fig. S1. PM_2.5_ mass concentration comparisons between the sampled filter and the nearest national monitoring station. (a): time-series plot, and (b) 1:1 plot**


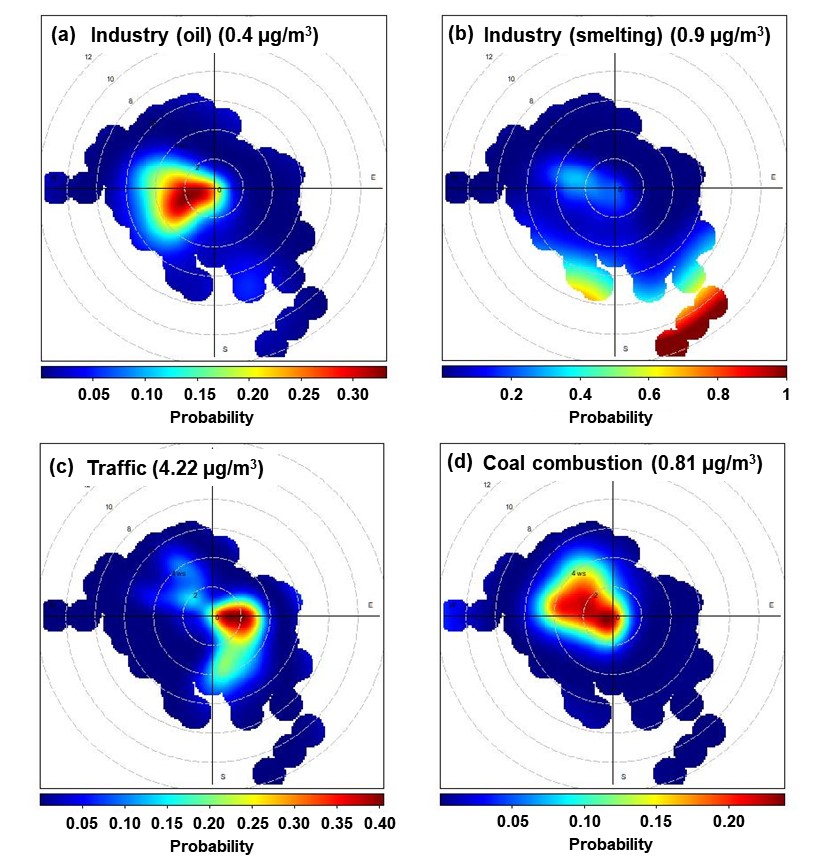


* The center of each figure is the measurement site

** The scale of the circle shows the wind speed (m/s)

Fig. S2 The CPF results of (a) industry (oil), (b) industry (smelting), (c) traffic, and (d) coal combustion sources


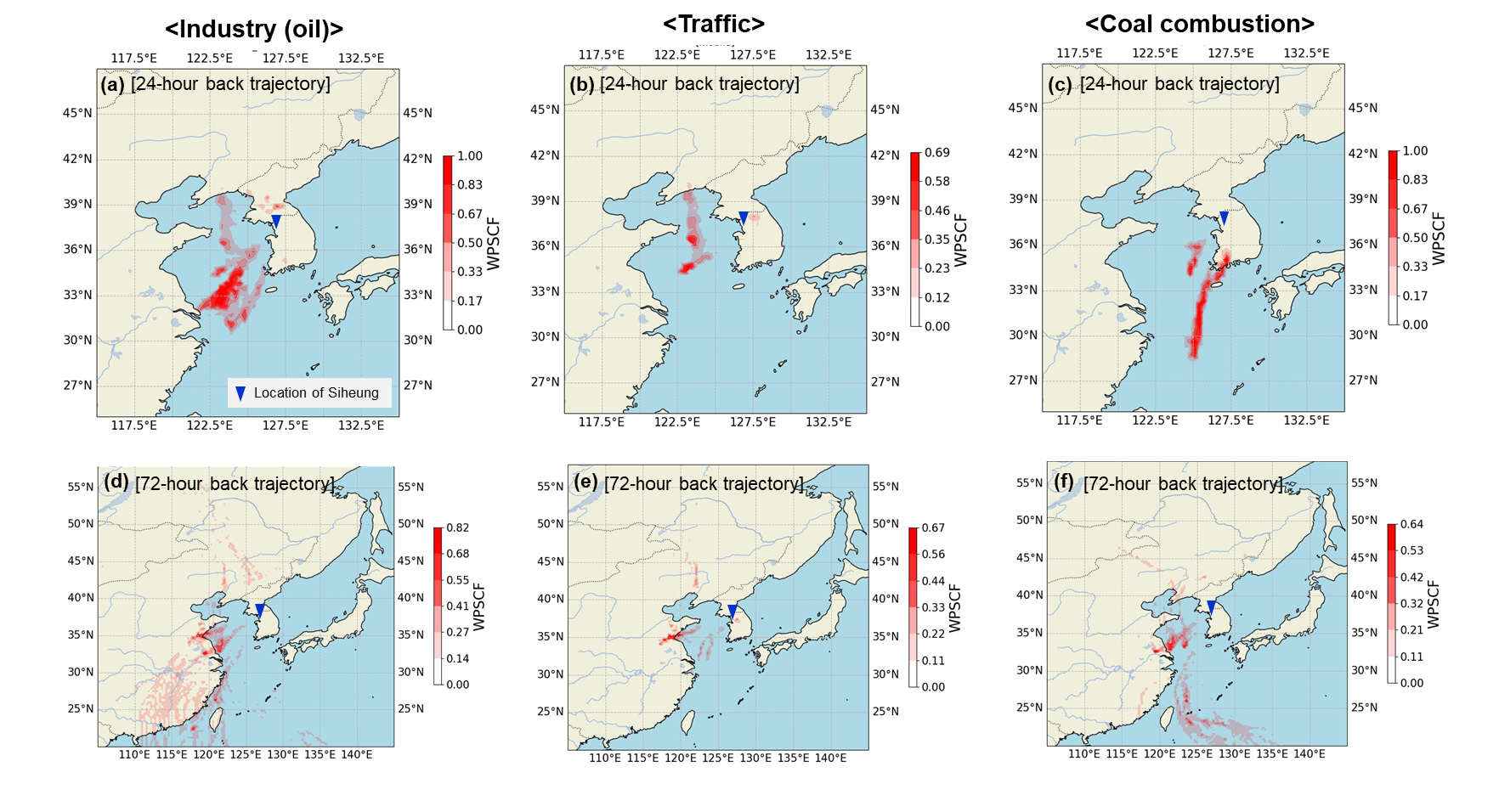


Fig. S3 PSCF results of PM_2.5_ sources in Siheung, Republic of Korea,
24-hour back trajectory of (a) Industry (oil); (b) Traffic; (c) Coal combustion,
74-hour back trajectory of (d) Industry (oil); (e) Traffic; (f) Coal combustion

**References**

Briffa J, Sinagra E, Blundell R (2020) Heavy metal pollution in the environment and their toxicological effects on humans. Heliyon 6:e04691. https://doi.org/10.1016/j.heliyon.2020.e04691

Fan MY, Zhang YL, Lin YC, et al (2021) Specific sources of health risks induced by metallic elements in PM2.5 during the wintertime in Beijing, China. Atmos Environ 246:118112. https://doi.org/10.1016/j.atmosenv.2020.118112

Hu X, Zhang Y, Ding Z, et al (2012) Bioaccessibility and health risk of arsenic and heavy metals (Cd, Co, Cr, Cu, Ni, Pb, Zn and Mn) in TSP and PM2.5 in Nanjing, China. Atmos Environ 57:146–152. https://doi.org/10.1016/j.atmosenv.2012.04.056

Kim S, Kim TY, Yi SM, Heo J (2018) Source apportionment of PM2.5 using positive matrix factorization (PMF) at a rural site in Korea. J Environ Manage 214:325–334. https://doi.org/10.1016/j.jenvman.2018.03.027

National Institute of Environmental Research (2019) Korean exposure factors handbook. Republic of Korea

Park M Bin, Lee TJ, Lee ES, Kim DS (2019) Enhancing source identification of hourly PM2.5 data in Seoul based on a dataset segmentation scheme by positive matrix factorization (PMF). Atmos Pollut Res 10:1042–1059. https://doi.org/10.1016/j.apr.2019.01.013

US EPA (2009) Risk Assessment Guidance for Superfund Volume I: Human Health Evaluation Manual (Part F, Supplemental Guidance for Inhalation Risk Assessment). Off Superfund Remediat Technol Innov Environ Prot Agency I:1–68

US EPA (2021) Regional Screening Levels (RSLs) Tables. https://www.epa.gov/risk/regional-screening-levels-rsls-generic-tables. Accessed 18 Aug 2021
